# *Spiranthes himalayensis* (Orchidaceae, Orchidoideae) a new species from Asia

**DOI:** 10.3897/phytokeys.89.19978

**Published:** 2017-11-14

**Authors:** Siddharthan Surveswaran, Pankaj Kumar, Mei Sun

**Affiliations:** 1 School of Biological Sciences, The University of Hong Kong, Pokfulam Road, Hong Kong S.A.R., China; 2 Centre for Ecological Sciences, Indian Institute of Science, Bangalore 560012, India; 3 Kadoorie Farm & Botanic Garden, Lam Kam Road, Lam Tsuen, Tai Po, New Territories, Hong Kong S.A.R., China

**Keywords:** Karnataka, Manipur, Orchid, *Spiranthes hongkongensis*, *Spiranthes nivea*, *Spiranthes sinensis*, Tamil Nadu, Yunnan, India, China

## Abstract

*Spiranthes himalayensis* is described here as a new species based primarily on molecular phylogenetic evidence followed by morphological comparison with other Asian *Spiranthes* species. It is distributed widely from southern India to tropical China. Phylogenetic analysis shows its close affinity to *S. nivea* which is endemic to Taiwan. Morphologically, the new species looks close to *S. sinensis* and *S. hongkongensis*. *S. himalayensis* is an allogamous species which can be differentiated from its allies on the basis of pubescent plant body, floral bract longer or of the same length as that of ovary, petals with blunt apex, labellum width around hypochile same as the width of epichile, epichile widely flabellate or semi-tunicate, column length equal to or more than 1.5mm, clavate operculum attached to the column on the broader part by an arm-like extension emerging from the upper part of column and a well developed rostellum partitioning the stigma and pollinarium.

## Introduction

*Spiranthes* Richard (1817:28) is a genus of terrestrial herbs belonging to the family Orchidaceae, comprising about 36 species, four varieties and four natural hybrids (Govaerts et al. 2017). The genus is widely distributed from Eurasia to Southwest Pacific, North Africa, North and Central America to the Caribbean (Govaerts et al. 2017). Of these, around seven species are found in Asia, including three in China and two in India (Chen et al. 2009, Zhou et al. 2016, Govaerts et al. 2017).

**Figure 1.**
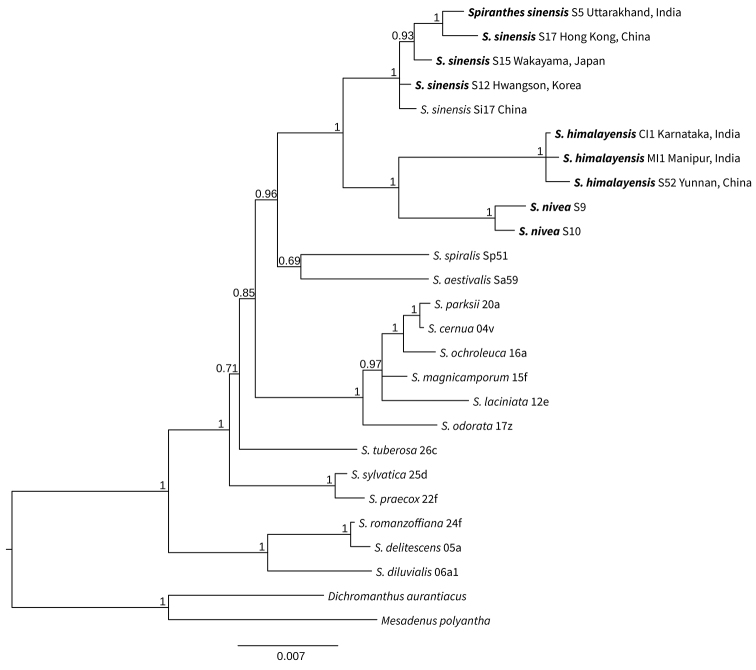
Bayesian phylogenetic tree indicating *Spiranthes himalayensis* Survesw., Kumar & Mei Sun sp. nov. Sequences generated for this study are indicated in bold, specimen identification numbers and area of collection are indicated beside the species names. Bayesian probability supports are indicated at the nodes.

While sampling *Spiranthes* in an aim to study the evolution and phylogeography of the genus in Asia, a white-flowered pubescent *Spiranthes* was discovered, which, based on DNA studies, was found to be distinct with respect to other known *Spiranthes* species (Fig. 1). It showed close affinity to *S. nivea* T.P Lin & W.M. Lin (Lin and Lin 2011) which is endemic to Taiwan (Fig. 1). It was also proven not to be *S. sinensis* (Pers.) Ames or *S. spiralis* (L.) Chevall. which are commonly distributed in Asia and Europe respectively (Fig. 1). Based on both morphological and genetic data, this white-flowered pubescent species is hitherto considered to be a new species that had formerly been misidentified as *S. sinensis*, *S. spiralis* or *S. hongkongensis* S.Y. Hu & Barretto in India and China (see Taxonomic notes). Based on sampling in southern India, North East India and South China, this species is now known to be widely distributed. Considering this as a new species, its morphological synapomorphies are enumerated here. Morphological comparisons have also been provided with allied species, *S. sinensis*, *S. hongkongensis* and *S. nivea*.

## Methods

Morphological observations of the new species were carried out based on living plants as well as dry specimens. Measurements were made using a ruler and a micrometer. Both herbarium and fresh specimens were examined under a stereo dissecting microscope and photographs were taken with a Nikon SMZ16 stereomicroscope connected to a digital camera.

A combined dataset of nuclear ITS, chloroplast *trnL-trnLF* intron/intergenic spacer, *trnS-trnG* intergenic spacer and maturase K (*matK*) sequences were used for the analysis (Table 1). The sequences were generated using primers described in Dueck et al. (2014). Molecular phylogenetic analysis was performed using MrBayes version 3.2.6 (Ronquist et al. 2012). One million generations of MCMC chains were run in MrBayes implementing the GTR+I+G model. Independent analysis of the nuclear and plastid markers were congruent and the combined analysis is presented here.

**Table 1.** Genbank accession numbers of sequences used.

Name	Specimen id	ITS	*trnL*	*trnSG*	*matK*
*Spiranthes sinensis*	S5	MF286487	MF286400	MF286356	MF286445
*S. sinensis*	S17	MF286496	MF286411	MF286367	MF286455
*S. sinensis*	S15	MF286494	MF286409	MF286365	MF286453
*S. sinensis*	S12	MF286493	MF286406	MF286362	MF286450
*S. sinensis*	Si17	KM262400	KM283611	KM283585	KM262488
*S. himalayensis*	C1 (HJCB 0442)	MF286478	MF286389	MF286343	MF286432
*S. himalayensis*	M1 (HJCB 1001)	MF286481	MF286391	MF286346	MF286435
*S. himalayensis*	S52	MF286511	MF286430	MF286387	MF286475
*S. nivea*	S9	MF286490	MF286403	MF286359	MF286448
*S. nivea*	S10	MF286491	MF286404	MF286360	MF286449
*S. spiralis*	Sp51	KM262410	KM283619	KM283595	KM262490
*S. aestivalis*	Sa59	KM262409	KM283621	KM283594	KM262492
*S. parksii*	20a	EU384861	EU384800	EU384739	KM262231
*S. cernua*	04v	KM262285	KM283636	KM283448	KM213798
*S. ochroleuca*	16a	KM262322	KM283680	KM283492	KM213844
*S. magnicamporum*	15f	KM262320	KM283678	KM283490	KM213843
*S. lacinata*	12e	KM262315	KM283672	KM283484	KM213829
*S. tuberosa*	26c	KM262384	KM283623	KM283568	KM262466
*S. sylvatica*	25d	EU384871	EU384819	EU384759	KM262464
*S. praecox*	22f	KM262363	KM283733	KM283545	KM262438
*S. romanzoffiana*	24f	KM262371	KM283741	KM283553	KM262447
*S. deliticens*	05a	KM283646	KM283646	KM283458	KM213774
*S. diluvialis*	06a1	KM262298	KM283649	KM283461	KM213777
*Dichromanthus aurantiacus*	–	AJ539485	AJ544468	–	AJ543913
*Mesadenus polyantha*	–	KU752294	KU740269	–	AJ543916

## Taxonomy

### Spiranthes himalayensis

Taxon classificationPlantaeAsparagalesOrchidaceae

Survesw., Kumar & Mei Sun sp. nov.

urn:lsid:ipni.org:names:77167300-1

**Figure 2.**
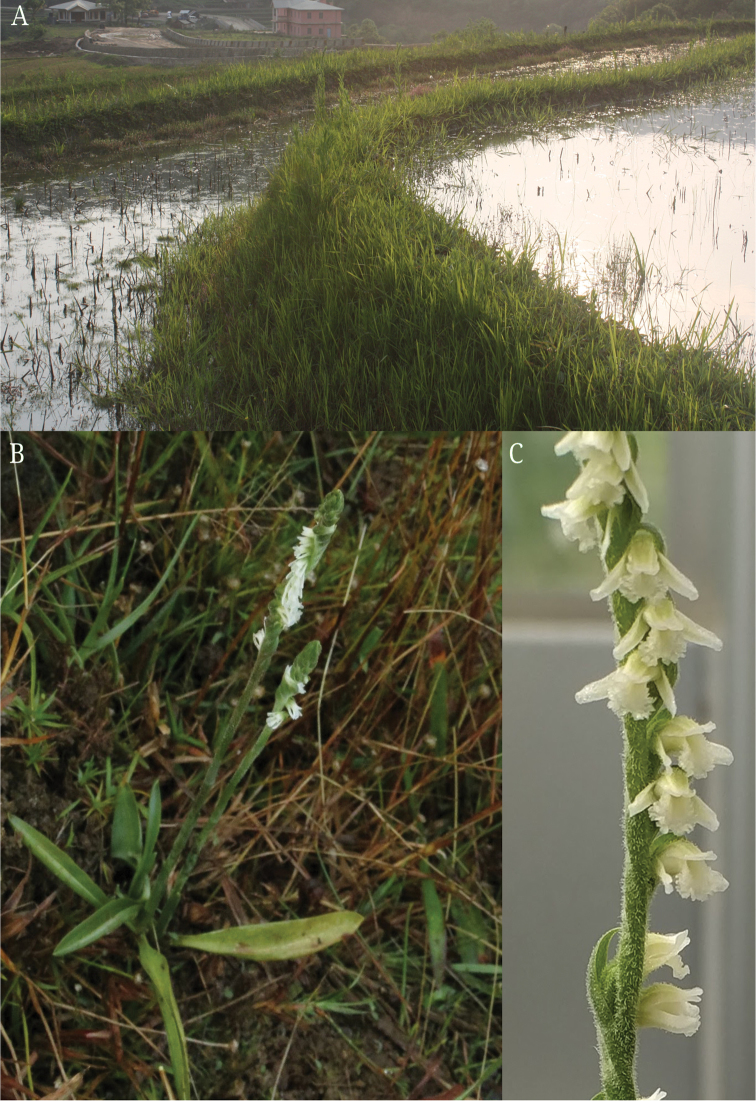
Habitat and habit of *Spiranthes himalayensis* Survesw., Kumar & Mei Sun, sp. nov. **A** Habitat (type location) - along the bunds of paddy fields **B** Habit of *S. himalayensis* **C** Close-up of the inflorescence showing white coloured flowers with densely covered glandular hairs.

**Figure 3.**
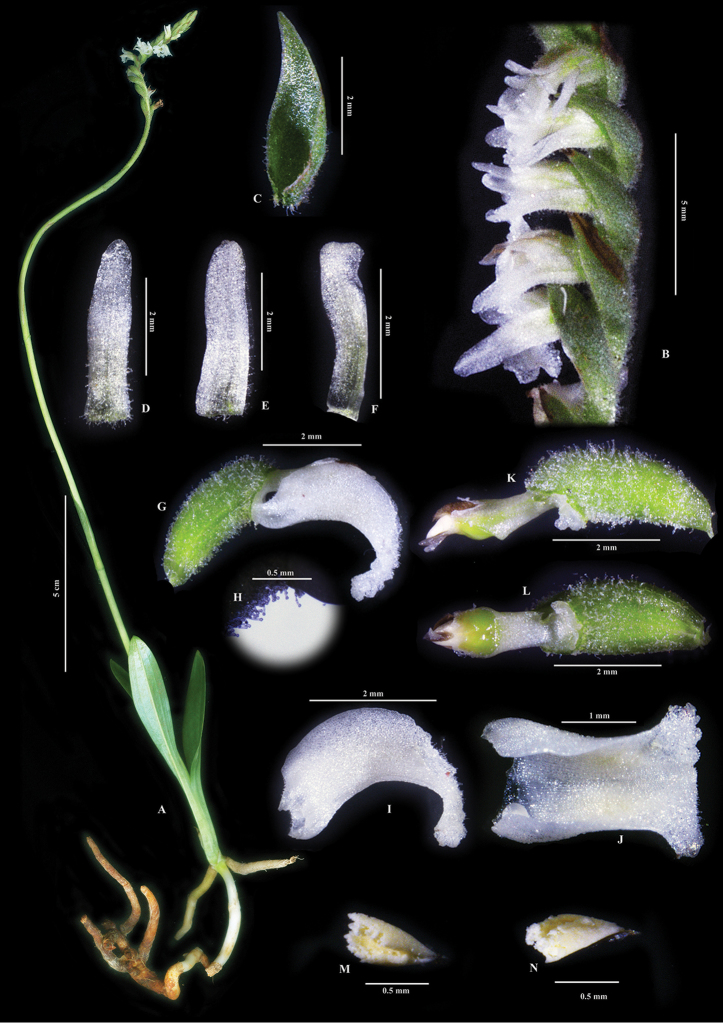
*Spiranthes himalayensis* Survesw., Kumar & Mei Sun, sp. nov. **A** Complete plant **B** Inflorescence **C** Floral bract **D** Dorsal sepal **E** Lateral sepal **F** Petal **G** Ovary with column and labellum **H** Close-up showing the glandular hairs on flower and ovary **I** Side view of labellum **J** Top view of labellum **K** Side view of ovary with column **L** Front view of ovary and column **M** Front view of pollinarium **N** Side view of pollinarium. Note: The pinkish/bluish hue on petals of *S. himalayensis* is due to the black background in the plates while the flowers appear fully white in natural light.

**Figure 4.**
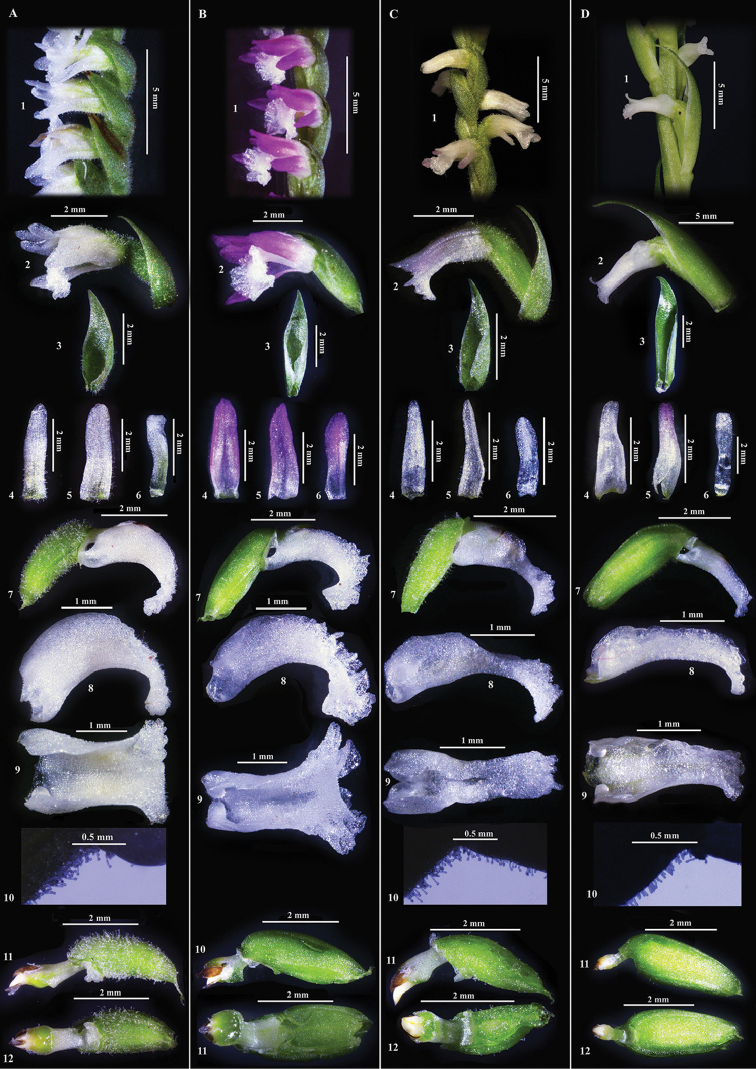
Comparison of morphological characters of allied species. **A***Spiranthes himalayensis*: **1** Close-up of inflorescence **2** A flower **3** Floral bract **4** Dorsal sepal **5** Lateral sepal **6** Petal **7** Ovary with column and labellum **8** Labellum (side view) **9** Labellum (top view) **10** Close-up showing glandular hairs on labellum and ovary **11** Ovary with column (side view) **12** Ovary with column (ventral view) **B***S. sinensis*: **1** Close-up of inflorescence **2** A flower **3** Floral bract **4** Dorsal sepal **5** Lateral sepal **6** Petal **7** Ovary with column and labellum **8** Labellum (side view) **9** Labellum (top view) **10** Ovary with column (side view) **11** Ovary with column (ventral view) **C***S. hongkongensis*: **1** Close-up of inflorescence **2** A flower **3** Floral bract **4** Dorsal sepal **5** Lateral sepal **6** Petal **7** Ovary with column and labellum; 8. Labellum (side view) **9** Labellum (top view) **10** Close-up showing glandular hairs on labellum and ovary **11** Ovary with column (side view) **12** Ovary with column (ventral view) **D***S. nivea*: 1. Close-up of inflorescence; 2. A flower **3** Floral bract **4** Dorsal sepal **5** Lateral sepal **6** Petal **7** Ovary with column and labellum **8** Labellum (side view) **9** Labellum (top view) **10** Close-up showing glandular hairs on labellum and ovary **11** Ovary with column (side view) **12** Ovary with column (ventral view). Note: The pinkish/bluish hue on petals of *S. himalayensis*, *S. nivea* and *S. hongkongensis* is due to the black background in the plates while the flowers appear fully white in natural light.

Figs 2B , 2C , 3 , 4A

#### Type.

INDIA. Manipur: Ukrul district, Imphal-Jessami road, found on a paddy field on the way to Ukhrul town from Imphal, 1 May 2016. *S. Surveswaran 1* (*HJCB 1001*) (holotype JCB!) (specimen id M1 in DNA based phylogeny, Fig. 1).

#### Diagnosis.

*Spiranthes himalayensis* Survesw., Kumar & Mei Sun *sp. nov*. is similar to *S. hongkongensis*, *S. nivea* and *S. sinensis*, but can be differentiated on the basis of its allogamous mode of reproduction from *S. hongkongensis* and *S. nivea* which are both autogamous. It can also be easily separated from *S. nivea* by its pubescent body. Other morphological distinctions separating this new species from *S. hongkongensis*, *S. nivea* and allogamous *S. sinensis* include: floral bract longer or of the same length as the ovary, petals with blunt apex, labellum width around hypochile is same as the width of epichile, epichile widely flabellate or semi-tunicate, column length equal to or more than 1.5 mm, clavate operculum attached to the column on the broader part by an arm-like extension emerging from the upper part of column and a well-developed rostellum separating the stigma from the pollinarium.

#### Description.

Terrestrial herbs with perennating rhizome. Plants c. 16–30 cm tall. Rhizome about 3mm in diameter. Stem erect, leaf clustered towards the base. Leaves 2 to 5 per plant, broadly linear or linear-lanceolate, 4.0–6.0 cm long, ca. 1.5cm wide, 3-veined with depression on upper surface and elevated lining underneath, spreading from the base of the stem. Inflorescence tall, up to 30 cm long, terete, pubescent, covered with glandular hairs, with 1–3 sterile bracts sheathing the peduncle, flowers spirally arranged, clustered towards the upper one-fourth of the peduncle with flowers opening from the base. Flowers widely open 0.22 × 0.26 cm wide, 0.43 cm long, pale white to pale butter-white, pubescent with glandular hairs. Bracts green, equal to or longer than the combined length of pedicel and ovary, ovate-lanceolate, 0.40–0.50 cm long, ca. 0.17 cm wide, acuminate, hairy on the outer surface, sheathing the base of flower, margin white. Dorsal sepal white, hairy towards the base, elongated-triangular, 0.35–0.40 cm long, ca. 0.10 cm wide, obtuse. Lateral sepals white, pubescent towards the base, obliquely elliptic, 0.37–0.40 cm long, 0.10 cm wide, obtuse. Petals white, pubescent towards the base, obliquely elliptic, ca. 0.37 cm long, ca. 0.07 cm wide towards the base, 0.09 cm wide towards apex, apex blunt. Labellum white, distinctly divided into hypochile and epichile with a constriction in the middle, hairy on the outer surface, ca. 0.50 cm long; hypochile concave, ca. 0.30 cm long, 0.33–0.34 cm wide, attached at the base of short foot below the column, saccate, sac 0.05 cm deep, with one semi-globular gland on each side (0.05 cm wide, 0.04 cm high), margin entire and raised upwards till the constriction; epichile semi-tunicate, flabellate, ca. 0.20 cm long, ca. 0.30 cm wide, margin undulate, slightly dentate with some papillose hairs on the front semi-tunicate part. Column green-white, obconical, quadrangular transversely quandrangular, 0.25 cm long, 0.05 cm at the base, 0.07 cm wide towards the apex, with a short foot at the base, 0.06 cm long, stigma at the apex on the lower side, green in colour, shiny, filled with viscid liquid, trapezoid shaped, broad towards base (0.08 cm), narrower towards apex (0.06 cm), 0.06 cm long. Rostellum well developed with two semi-transparent, clavate, rostellar arms projecting in the front above stigma, 0.06 cm long. Pollinarium yellow, ovate with a deep cleft, 0.22 cm long, 0.09 cm wide, narrower end at the apex while the lobes inside covered with operculum, with a ligulate viscidium at the narrower end, 0.1 cm long. Operculum yellow-brown coloured, partly embedded on the upper part of column, not free, convex, ca. 0.10 cm long, 0.06 cm wide. Ovary sessile or with inconspicuous pedicel, densely haired, fusiform, 0.20–0.25 cm long, 0.11–0.13 cm wide. Fruits obliquely clavate, ca. 0.30 cm long, 0.12 cm wide, densely pubescent with glandular hairs.

#### Phenology.

Flowering: March–June. Fruiting: March–July.

#### Habitat.

Marshy areas near mountain streams or on bunds of paddy fields (Fig. 2A) where water is stagnant. Plants were usually found growing on clayey soil along with grasses.

#### Etymology.

The specific name refers to the mighty Himalayan mountain range which is an important geographical feature in Asia. The samples collected for this study were not from the Himalayas. However, based on herbarium records and communication with other researchers, it is believed that this species is widespread in the lower altitudes of Himalayas. The evolutionary origin of this species and other Asian *Spiranthes* are to be further elucidated.

#### Currently known locations of distribution.

India (Karnataka, Manipur & Tamil Nadu) & China (Yunnan and most likely Hainan (see taxonomic notes for details)).

#### Conservation status.

Based on DNA analysis, the occurrence of *Spiranthes himalayensis* can be confirmed in India and China. This species is likely to be present in a much wider geographic range where it has long been misidentified (see taxonomic notes). It was not possible to assess the specimens which might have been misidentified as *S. sinensis* or *S. spiralis* across its broad distributional range. It is emphasised that the type specimen was collected in an agricultural field and other specimens were found on roadsides along mountain slopes above 1000 m in its wide distributional range. Therefore it is speculated that this species might be of least concern for conservation. However large-scale habitat loss and fragmentation of habitats in Asia might pose a threat to this species. Since a thorough assessment has not been undertaken, the conservation status of Not Evaluated (NE) has been assigned to this species as per IUCN Red List categories and criteria (2017).

#### Other specimens examined.

INDIA. Manipur: Ukrul district, Imphal-Jessami Road, found on paddy fields on the way to Ukhrul town, 1200 m, 1 May 2016, *S. Surveswaran 1HJCB 1001* (M1-DNA specimen) (JCB); Karnataka: Chickmagalur district, near Kemmankundi, 1400 m, 14 June 2015, *S. Surveswaran 4* (*HJCB 0442*) (C1-DNA specimen) (JCB!); Tamil Nadu: Coimbatore, near Konalar anti-poaching protection camp, Grass Hills, Valparai Range, Anaimalai Tiger Reserve, 1200 m, 13 March 2012, *K. Ravikumar & A.C. Tangavelou FRLH-121312* (FRLH!). CHINA. Yunnan Province: Malipo County, Wenshan, near Xiajinchang village, 1400 m, 20 May 2016, *S. Surveswaran S52*.

#### Note.

Hairy *Spiranthes* have been reported very often from India, sometimes under the name of *S. sinensis* (Kumar and Manilal 1994, Misra 2004) and, at other times, under the name of *S. spiralis* (Deva and Naithani 1986, Misra 2007). Hooker (1890) also described two species of *Spiranthes*, *S. australis* (= *S. sinensis*) and *S. autumnalis* (= *S. spiralis*) from India. As the protologue of *S. sinensis* does not mention the presence or absence of hairs (Persoon 1807), there has been no clear distinction between *S. sinensis* and the new white flowered *S. himalayensis* amongst the various reports. So far, a true *S. spiralis* has not been found in India and the specimens treated as *S. spiralis* might have been *S. himalayensis* sp. nov. In this study, it was out of the scope to trace all specimens or vouchers of hairy *Spiranthes* and distinguish them from *S. sinensis* which could also be hairy in some natural populations.

Seidenfaden (1978) reported *Spiranthes sinensis* from Thailand, illustrating a hairy plant with long and stalked glands in the hypochile. This specimen is also suspected to be the new species *S. himalayensis* described here. Deva and Naithani (1986) reported *S. sinensis* and *S. spiralis* from northwest Himalaya, differentiating them on the basis of multiple roots; elongated, fleshy, cylindrical tubers; lanceolate leaves, present during flowering and pink flowers in the former against 2–3 roots; fleshy carrot shaped tuber; ovate leaves, absent during flowering; white or greenish-white flowers in the latter. They also illustrated *S. sinensis* with hairy flowers and well developed rostellum with two rostellar arms. For *S. spiralis*, they just gave an illustration of tubers, but we believe that this sample could also be the new species described here. They also mentioned that *S. sinensis* was found in two colours, pink and white. This could be the source of confusion leading to the misidentification of our new species as *S. sinensis* by other workers. Kumar and Manilal (1994) reported *S. sinensis* from Kerala, South India, stating that it is a variable species but no description was provided. Pink flowered forms of *Spiranthes* have not been observed in southern India, indicating an absence of the true *S. sinensis* in this area and the white flowered species reported as *S. sinensis* is also most likely to be the new species, *S. himalayensis*, described here. Acharya et al. (2010) recently described a new record of *S. spiralis* from Pokhara valley in Nepal with glandular hairs and a flowering season similar to *S. himalayensis*. It is suspected that this plant from Nepal could also be *S. himalayensis* sp. nov. but it was not possible to acquire the specimen for detailed morphological or DNA study. The morphological description in this publication would help with the correct identification of the new species, *S. himalayensis* in the future.

Pridgeon et al. (2003) (fig. 203.1) illustrated *Spiranthes sinensis* based on *Pantling 107* (K) and *Grierson & Long 3986* (K), showing hairy plants looking very similar to *S. himalayensis* sp. nov. However, the flower colour cannot be confirmed from the line drawings.

Huang et al. (2014) reported the presence of a species of *Spiranthes* at an altitude of about 800 m on a grassy slope of Diaoluoshan of Hainan Island, China, which they revised as *S. hongkongensis* from a previous misidentification as *S. sinensis.* However, based on the plant picture presented in Hainan Rainforest Exhibition Hall, the Hainan species looks like the new species *S. himalayensis* (based on floral morphology and lack of fruit set at the base of inflorescence, personal observations). Therefore it is likely that *S. himalayensis* might be extending its range up to Hainan Island. Genetic evidence of the occurrence of *S. himalayensis* in China has been obtained from a population located in Yunnan where at least two plants were consistently placed in the species clade of *S. himalayensis* whether nuclear ITS or chloroplast DNA was used in the data analysis (Surveswaran and Sun, unpublished).

Based on DNA studies (Fig. 1), the samples of *Spiranthes himalayensis* formed a distinct species clade, sister to *S. nivea* clade. Both *S. himalayensis* and *S. nivea* are distinct from *S. sinensis* which forms a large monophyletic clade including samples from a wide geographical range such as Hong Kong, Taiwan and mainland China, Korea, Japan, Australia and New Zealand. It should be noted that *S. sinensis* is highly polymorphic in flower colour, varying from purple pink to white and also in the extent of hairiness, from glandular to glabrous. The distinguishing morphological features of *S. sinensis* include: floral bract equal to or longer than the length of ovary, petals with rounded apex, labellum width around hypochile is half of the width of epichile, epichile widely semi-tunicate, column length less than 0.5 mm long, upper part of operculum attached to column with the arm emerging from the top of column, well developed rostellum partitioning the stigma and pollinarium. On the other hand, *S. hongkongensis* always has white flowers with occasionally pinkish tint on the petal and glandular hairs on various parts of the plant, but its floral bract is equal to or shorter than the length of ovary, petals have rounded apex, labellum width around hypochile is equal or slightly greater than the width of epichile, epichile not tunicate, column length up to 1mm long, lower half of operculum embedded on to column, rostellum absent and pollinia lying directly above stigma rendering autogamy. In contrast, *S. nivea* has short papillose hairs inconspicuous to the naked eye, flower colour is always white without pinkish tint, floral bracts are much longer than the length of ovary, petals have a blunt apex, labellum width around hypochile being more than the width at epichile, epichile not being tunicate, column length almost 0.5 mm, lower half of operculum embedded in the column, rostellum absent and pollinia lying directly above stigma rendering autogamy (see Table 2).

**Table 2.** Comparison of morphological characters of *Spiranthes himalayensis* Survesw., Kumar & Mei Sun, sp. nov., *S. sinensis*, *S. hongkongensis* and *S. nivea.*

Character	*Spiranthes himalayensis*	*Spiranthes sinensis*	*Spiranthes hongkongensis*	*Spiranthes nivea*
**Flowering time**	March to June	April to June (in Hong Kong), August to September (in India)	March to early June	April to June
**Inflorescence**	Pubescent with glandular hairs	Pubescent or sometimes glabrous	Pubescent with glandular hairs	Glabrous, but with microscopic non-glandular papillose hairs
**Flower**	White, pale white, widely open, 0.4–0.5 × 0.15 cm wide, ca. 0.43 cm long	Pink, or purple, but occasionally white, widely open, 0.19 × 0.22 cm wide, ca. 0.45 cm long	White, petals and sepals rarely pink tinged, labellum white, widely open, 0.10, 0.12 cm wide, ca. 0.38 cm long	White, tubular on lower 2/3rd, open towards the tip, 0.09 × 0.09 cm wide, ca. 0.41 cm long
**Bract**	Equal to or longer than ovary, 0.4–0.5 cm long, ca. 0.17 cm wide	Equal to or longer than ovary, 0.36 cm long, 0.14 cm wide	Equal to or shorter than ovary, ca. 0.37 cm long, 0.19 cm wide	Much longer than ovary arching over the flower, 0.49 cm long, 0.16 cm wide
**Petal**	Apex blunt	Apex rounded	Apex rounded	Apex blunt
**Labellum**	Labellum white, pubescent on the outer surface, distinctly divided into hypochile and epichile with a constriction in the middle, ca. 0. cm long; hypochile ovate, concave or saccate, ca. 0.30 cm long, 0.33–0.34 cm wide, sac 0.05 cm deep, with a pair of semi-globular gland at the base (0.05 cm wide, 0.04 cm high), margin entire and raised upwards till the constriction, two broad pale green patch at the constriction; epichile semi-tunicate, flabellate, ca. 0.20 cm long, ca. 0.30 cm wide, margin undulate, slightly dentate with some papillose hairs on the front semi-tunicate part	Labellum pink or white, glabrous, not distinct into epichile and hypochile due to lack of constriction, rather a semi-cylindrical basal portion with tunicate or flabellate frontal portion, 0.46 cm long; basal portion 0.15 cm wide, 0.23 cm long, saccate, sac 0.03 cm deep, margin raised with a pair of semi-globular glands at the base (0.05 cm wide, 0.05 cm high), margin smooth towards base, undulate from almost middle of the basal portion to the whole length of frontal portion; frontal portion semi-tunicate or flabellate, 0.13cm long, 0.16 cm wide at base, 0.35 cm wide towards the apex, margin undulate, slightly dentate	Labellum white with rare pinkish tinge, distinctly divided into hypochile and epichile with a constriction in the middle, ca. 0.58 cm long; hypochile ovate, slightly concave or saccate, 0.26 cm long, 0.32 cm wide, 0.06 cm deep, with a pair of trapezoidal shaped gland at the base (0.09 cm wide, 0.07 cm high), margin entire and raised upwards till the constriction; no colour patch at the constriction; epichile semi-tunicate, flabellate, ca. 0.26 cm long, ca. 0.26 cm wide, margin undulate, slightly dentate, glabrous	Labellum white, glabrous, not distinct into epichile and hypochile due to lack of constriction, rather a semi-conical basal portion with semi-cylindric frontal portion, 0.43cm long; basal portion 0.24 cm wide towards base, 0.14 cm wide towards upper part, 0.24 cm long, saccate, sac 0.03 cm deep, margin raised with a pair of semi- globose glands at the base (0.04 cm wide, 0.04 cm high), margin smooth till the base of frontal part of labellum; frontal portion not widely spreading, margin convolute, 0.19 cm long, 0.16 cm wide at base, 0.14 cm wide towards the apex, margin undulate towards base and slightly dentate towards apex
**Labellum apex**	Recurved, > 270 degrees	Strongly recurved, almost 360 degrees	Recurved by < 270 degrees	Recurved by < 270 degrees
**Column**	Column, obconical, 0.25 cm long, 0.05 cm at the base, 0.07 cm wide towards the apex; foot 0.06 cm long; stigma trapezoid shaped, broad towards base (0.08 cm), narrower towards apex (0.06 cm), 0.06 cm long.	Column conical, 0.23 cm long, 0.08 cm wide at the base, 0.10 cm wide at the apex; foot 0.05 cm long; stigma almost semicircular in shape, 0.09 cm wide	Column, obconical, 0.21 cm long, 0.07 cm at the base, 0.09 cm wide towards the apex; foot 0.05 cm long; stigma crescent or semicircular shaped, 0.08 cm wide	Column obconical. 0.12 cm long, 0.04 cm wide at base, 0.05 cm wide towards apex; foot 0.04 cm long; stigma semicircular in shape, 0.05 cm wide
**Operculum attachment on column**	An inconspicuously separating arm on the top of column attached to the front broader part of operculum	An extension from the upper part of column protruding out and attached to the top of operculum	Broader front of operculum embedded in the column (no extension projecting from column on to operculum)	Almost half of the broader part of operculum embedded on to the top of the column (no extension projecting from column on to operculum)
**Rostellum**	Well developed with two semi-transparent, clavate, rostellar arms projecting in the front above stigma, 0.06 cm long.	Well developed with two semi-transparent, clavate, rostellar arms projecting in the front above stigma, 0.05 cm long	Not visible or absent	Not visible or absent
**Pollination**	Allogamous	Allogamous	Autogamous (due to lack of rostellum, pollinia falls on the stigma on its own)	Autogamous (due to lack of rostellum, pollinia falls on the stigma on its own)

Based on preliminary chromosome counts, *Spiranthes himalayensis* and *S. nivea* both have a diploid chromosome number of about 30, similar to *S. sinensis.* It has been confirmed that *S. hongkongensis* is an allotetraploid (4× = 60), with *S. sinensis* as its maternal progenitor (Sun 1996, Chen and Sun 1998) and most likely the new species *S. himalayensis* as its paternal progenitor (Surveswaran and Sun, unpublished). *S. hongkongensis* was not included in the phylogeny because it is a well-known allotetraploid (Sun 1996) and sequencing of cloned ITS sequences of *S. hongkongensis* to identify the paternal and maternal progenitors of this species has been performed (Surveswaran and Sun, unpublished).

## Supplementary Material

XML Treatment for Spiranthes himalayensis
